# Assessing the validity and reliability of the Indonesian version of Patient-Reported Outcomes Measurement Information System (PROMIS) Global Health Scale v1.2

**DOI:** 10.1186/s12955-025-02336-4

**Published:** 2025-01-31

**Authors:** Vitriana Biben, Farida Arisanti, Efi Fitriana, Erika Maklun, Vindy Margaretha Miguna, Nabilla Fikria Alviani

**Affiliations:** 1 Physical Medicine and Rehabilitation Department, Faculty of Medicine, Universitas Padjadjaran/Dr. Hasan Sadikin General Hospital, Bandung, West Java Indonesia; 2 Faculty of Psychology, Universitas Padjadjaran, Bandung, West Java Indonesia

**Keywords:** Patient-reported outcomes, Quality of life, Questionnaire, Validity, Reliability

## Abstract

**Background:**

The assessment of Health-Related Quality of Life (HRQoL) is essential in clinical outcomes, focusing on the subjective perception of individuals regarding the physical, mental, and social aspects of health status. However, conducting a large-scale HRQoL assessment poses various challenges that necessitate the development of a non-burdensome instrument. One promising solution is adapting Patient-Reported Outcomes Measurement Information System (PROMIS) Global Health Scale v1.2 through translation, validation, and cross-cultural testing for non-English populations. Therefore, this study aimed to evaluate the validity and reliability of the Indonesian version of PROMIS Global Health Scale for comprehensive HRQoL assessment.

**Method:**

This cross sectional study involved a total of 343 participants, comprising patients, caregivers, and residents of Physical Medicine and Rehabilitation. PROMIS Global Health Scale v1.2 was subjected to translation and cultural adaptation using the Functional Assessment of Chronic Illness Therapy (FACIT) method. Content validity was tested by five experts using the Scale-Content Validity Index (S-CVI), and structural validity was evaluated through Confirmatory Factor Analysis (CFA). Internal consistency and test–retest reliability were examined using Cronbach's Alpha and intraclass correlation coefficient (ICC), respectively.

**Result:**

The Indonesian version of PROMIS Global Health Scale v1.2 showed strong validity and reliability. Content validity analysis produced a S-CVI/Universal Agreement of 0.90 with item analysis factor loading’s of > 0.3. Structural validity results were χ2/df (1.53), RMSEA (0.04), RMR (0.03), and CFI (0.99). The reliability results showed that Cronbach's Alpha for Global Physical Health (GPH) and Global Mental Health (GMH) was 0.61 and 0.77, respectively. Test–retest reliability assessment performed using intraclass correlation coefficients generated values of 0.72 for GPH (95% CI, [0.65,0.78])and 0.70 (95% CI [0.63,0.76]) for GMH.

**Conclusion:**

The Indonesian version of PROMIS Global Health Scale v1.2 showed sufficient content validity, structural validity, internal consistency, and reliability, which supported the application of this tool for HRQoL assessment in clinical and research settings.

## Background

Health is an essential determinant playing a significant role in shaping the quality of life (QoL) of individuals. According to World Health Organization (WHO), QoL is defined as “the perception of individuals regarding the position occupied in life in terms of culture and value systems, as well as goals, expectations, standards, and concerns” [1]. Several studies reported this context to have a multifaceted nature, among which the aspects associated with health are called Health-Related Quality of Life (HRQoL). In addition, HRQoL focuses on the subjective perception of the physical, mental, and social aspects of health status. This is considered the best outcome of medical interventions due to the ability to comprehensively assess the subjective perception and expectations of patients, [2, 3] including levels of satisfaction and feelings of worth exceeding physical well-being [4].

Several studies have been conducted recently to develop HRQoL measurement instrument that alleviates administrative burdens, particularly for large-scale reports and examinations. A promising instrument proven to be effective is Patient-Reported Outcomes Measurement Information System (PROMIS) Global Health Scale funded by the National Institute of Health. This is a questionnaire consisting of 10 questions covering various facets, such as general health, general QoL, physical and mental well-being, satisfaction with social activities, ability to engage in social activities, daily physical activity, emotions, fatigue, and pain [5, 6]. The completion time requires only 2 min, and several reports show that the use of PROMIS Global Health Scale v1.2 offers additional advantages by leveraging items response theory (IRT), where items are arranged on a scale (metric) according to the degree of 'difficulty' [7]. Consequently, this questionnaire has gained recognition as an assessment included in the standard set for adult general health by the International Consortium of Health Outcomes Measurement (ICHOM) [8].

HRQoL measurement instrument has been developed worldwide in the last decade, but a significant portion is predominantly available in the English language. The use of this instrument among non-English speaking populations necessitates various processes, including translation, validation testing, and cross-cultural reliability. PROMIS Global Health Scale v1.2 was translated into Dutch-Flemish, Norwegian, and Korean, with positive validity and reliability test results [7]. Despite the questionnaire being widely available in different languages, it is not yet translated into Indonesian.

An essential asset in the context of medical intervention and rehabilitation is a measurement instrument that ensures validity and reliability while alleviating administrative burdens. Among the populations in Indonesia with a diverse cultural and linguistic landscape, a culturally sensitive instrument is needed to effectively assess and monitor the impact of medical intervention. Therefore, this study aimed to evaluate the validity and reliability of the Indonesian version of PROMIS Global Health Scale v1.2 questionnaire, popularly known for the comprehensive assessment of various aspects of QoL. The results are expected to provide healthcare professionals with a robust and accessible instrument to facilitate precise evaluation and monitoring for enhancement of the quality of care and rehabilitation outcomes.

## Method

**Fig. 1:**
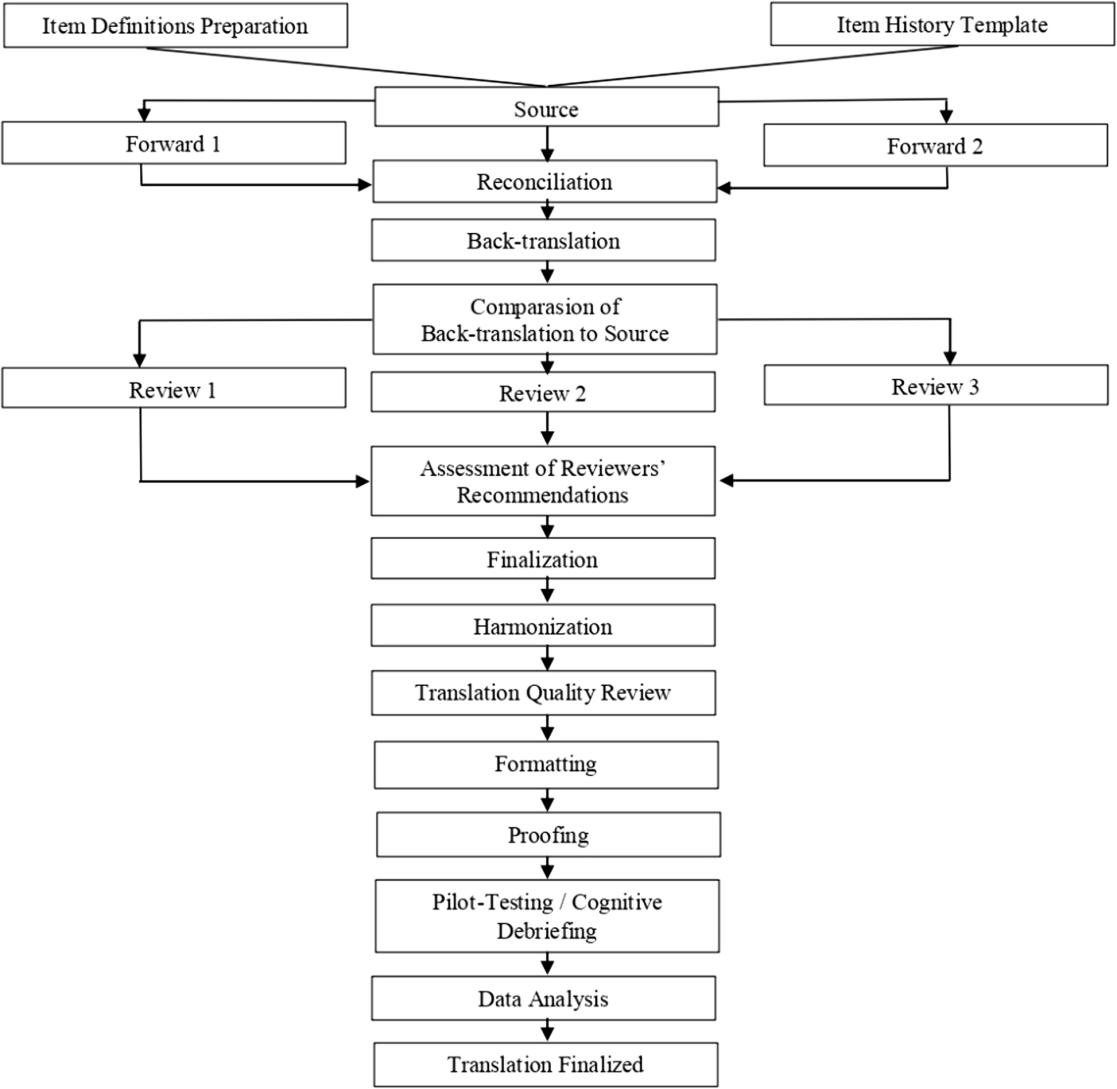
The FACIT translation method [9]

A cross-sectional design was used in this study to assess the validity and reliability of questionnaire items translated and culturally adapted into the Indonesian language using the Functional Assessment of Chronic Illness Therapy (FACIT) method (Fig. 1). Furthermore, participants were recruited from Physical Medicine and Rehabilitation Department Hasan Sadikin General Hospital located in West Java, Indonesia with consecutive sampling method using predetermined inclusion criteria, namely (1) age 19 years or older, (2) ability to understand instructions, (3) capability to proficiently speak, read, and write Indonesian, (4) independence, and (5) willingness to engage in the investigation procedures. All individuals were screened using MMSE, those with a cognitive issue (MMSE < 24) were excluded. Another criterion for exclusion was the existence of uncorrectable visual and hearing impairments. Presence of pre-existing medical condition was noted but not included in the data analysis due to the nature of the questionnaire.

Permission for translation and validation was provided by HealthMeasures, while the FACIT method was used in all translations of adult and pediatric PROMIS items. This process was performed in compliance with the guidelines recommended by the FACIT Translation Methodology to translate the PRO instrument, and the certified translation was approved by PROMIS Translation Director.

The stages of the translation process are as follows:

1. The study team initially prepares PROMIS Global Health scale and requests permission to conduct the translation process by sending electronic mail to the author of the original questionnaire.
2. Forward translations: The translation from the source to the target language is carried out by two independent professional translators who are native speakers of the target language.
3. Reconciliation: A third independent translator who is a native speaker of the target language reconciles the two forward translations by selecting one or creating a new version.
4. Back translation: The reconciled version is then back-translated by a native English-speaking translator with fluency in the target language. The translator avoids checking the original English items or the definition of each item, and the back translation into English must reflect the target language translation.
5. Back-translation review: The Translation Project Manager (TPM) compares the source English version and the back translation to identify differences as well as provide clarification to the reviewers about the intent behind the points. This particular step also leads to an initial assessment of harmonization between languages.
6. Expert Review: Three experts who are native speakers of the target language, independently check all previous steps and select the most appropriate translation for each item or provide an alternative translation in case the previous is unacceptable. These reviewers are often linguists or health professionals, specifically combined groups are recommended.
7. Pre-finalization Review: The TPM evaluates the comments of reviewers, identifies potential problems in the recommended translation, as well as formulates questions and comments to guide the language coordinator for the target language.
8. Finalization: The Language Coordinator, a native speaker of the target language, determines the final translation by reviewing all information in the item history and responding to comments of the TPM. An explanation of the final translation choice and justification is provided for the decision taken in case the final translation differs from the reconciled version or from what the individual reviewer recommended.
9. Harmonization and quality assurance: The TPM performs an initial assessment of the accuracy and equivalence of the final translation by comparing the final back translation with the source, then verifying that the decision-making process documentation is complete. A quality review conducted by PROMIS Statistics Center will address consistency with previous translations and other languages ​​where applicable, as well as between items. Furthermore, the Language Coordinator may be consulted again for any necessary additional input.
10. Formatting, typesetting, and proofreading of the final questionnaire or item form by two proofreaders working independently, and reconciliation of proofreading comments.
11. Cognitive testing and linguistic validation: The target language version is tested with native speakers and cognitive interviews are conducted in the target country by at least 5 participants to verify that the meaning of the items in the instrument is equivalent to the English source after translation.
12. Analysis of participant comments and finalization of translation: The TPM collects participant comments (translated back into English) and summarizes the stated problems. This Manager also verifies the solution proposed by the language coordinator to be consistent with the source and other languages.

A total of 10 participants with an age range of 18–57 years old and low to high education level were included in the cognitive debriefing process performed during this study. Furthermore, the word “global health” was found to be less understood, leading to using the term “general health” in the translated version. Most participants did not understand the definition of QoL, and only 1 out of 10 in the cognitive debriefing responded with “self-satisfaction” where the fulfillment of basic needs was perceived as life satisfaction. Subsequently, the paper-based translated questionnaire was administered to a sample population in the Medical Rehabilitation outpatient clinic at Dr. Hasan Sadikin Hospital from January to December 2023.

The investigation conducted by Hoogland, Boomsma, and Kline [10] reported a minimum sample size of 200. Another determination could be attained using the rule of thumb proposed by Nunnally, namely providing 10 samples for each indicator variable. This study applied a sample size of 200, which was the largest minimum size identified based on the literature reviewed. Only 10% of the 200 were added to anticipate incomplete or missing data, hence, the minimum total sample was 220.

Descriptive analysis was performed by presenting the demographic data of participants accompanied by an assessment of the mean, standard deviation, and subset scores. T-score was calculated for Global Physical Health (GPH) and Global Mental Health (GMH).

The collection of valid evidence was conducted as follows:

- *Content validity:* Relevance was assessed through judgment by Physical Medicine and Rehabilitation Specialists and linguistic experts who mastered the theoretical basis of the constructs used. These included 5 specialists from the Musculoskeletal, Neuromuscular, Cardiorespiratory, Geriatrics, and Pediatrics divisions at Dr. Hasan Sadikin Hospital. Furthermore, the expert reviewers were asked to assess the relevance of the question items based on the definitions proposed by PROMIS. The content validity index (CVI) value was determined using the relevance rating from the experts [11]. For item-CVI assessment (I-CVI), experts were asked to provide a relevance rating for each item using a scale of 1–4 where 1 = not relevant, 2 = somewhat relevant, 3 = quite relevant, and 4 = very relevant. Subsequently, each item was assessed for I-CVI, which was obtained by dividing the number of experts who provided a rating of 3–4 by the total experts, known as the proportion of agreement on the relevance. The next assessment was the CVI for the entire scale known as S-CVI, which was calculated using universal agreement and conservative method. The universal agreement was evaluated by experts (S-CVI/UA) through division of the number of I-CVI worth 1 by the total items. A more conservative way was to calculate the mean of I-CVI (S-CVI/Ave), where a good S-CVI number was above 0.7, and 0.8 was recommended for the new measurement instrument [12].
- *Structural validity:* This was conducted using Confirmatory Factor Analysis (CFA) to examine the internal structure of the Indonesian version of PROMIS Global Health Scale v1.2. The form of the structural model analyzed was a first-order factor with two correlated dimensions, and the estimation method applied was maximum likelihood. Additionally, CFA was used to determine the goodness of fit of the model, with the chi-square test showing a value near 0 which signified a minimal difference between implied and observed covariance matrices. The probability level should be > 0.05 as the chi-square value approached 0, and the model was considered fit when the chi-square to the degree of freedom ratio (χ2/df) = 3:1. Furthermore, Comparative Fit Index (CFI) with a score ≥ 0.95 represented acceptable model fit and Root Mean Square Error of Approximation (RMSEA) ≤ 0.08 denoted good fit.
- *Internal consistency:* Cronbach’s Alpha was used to measure internal consistency, and the coefficient was considered sufficient when equal to 0.6 or higher [13]. Additionally, item analysis was conducted to determine the consistency between item scores and the total score. This consistency could be observed from the large correlation coefficient between each item and the total score. Item correlation of < 0.30 represented poor discriminating power, while correlation ≥ 0.30 showed good discriminating power [12, 14]. The item analysis was also accompanied by Cronbach's Alpha internal consistency reliability value when an item was removed from the measurement. An increase in the alpha coefficient after removing an item from the total score signified that the item contributed less to the internal consistency. Meanwhile, a decreasing coefficient compared to the total score showed good internal consistency of an item [14].

*Test–retest reliability:* Test–retest was conducted on the same sample using the same PROM twice. Patients completed both paperbased tests independently either at the hospital or at home, with a 7-day interval between them with supervision of the researcher to minimize possibility of missing data. The researcher provided oral instructions on how to complete the test and asked patients to carefully read the instructions. Reliability was measured with intraclass correlation coefficient (ICC) with 95% confident intervals based on a mean-rating (k = 2), consistency, 2-way random-effects model to determine how well result can be distinguished from each other, despite measurement errors. ICC values less than 0.5 indicate poor reliability, values between 0.5 and 0.75 indicate moderate reliability, values between 0.75 and 0.9 indicate good reliability, and values greater than 0.90 indicate excellent reliability [15].

The data collected were analyzed using the Statistical Program for Social Sciences (SPSS Inc, Chicago, IL) version 26.0 for Windows and LISREL 10.3 software. To address missing data, multiple imputation statistical methods were employed to handle any missing values [16]. Moreover, this study was conducted after receiving approval with number sDP.04.03//X.2.2.1/3825/2023 from the Research Ethics Committee of Dr. Hasan Sadikin Hospital. All data supporting the results are available in the study paper and the supplementary information.

## Result

A total of 343 participants who completed PROMIS-GH v1.2 questionnaire were used as the sample population with demographic characteristics presented in Table 1. The participation rate was found to be 100%, but only 236 participants were re-tested in this study due to loss of follow-up.

**Table 1:** Study population

	Category	Number (subject)	Percentage (%)
Gender	Women	226	66.10
	Men	117	33.90
Age group	18–24 years	52	15.16
	25–34 years	93	27.11
	35–44 years	49	14.28
	45–54 years	74	21.57
	55–64 years	49	14.28
	65–74 years	21	6.12
	≥ 75 years	5	1.45
Education	Base	27	7.90
	Intermediate	174	50.60
	High	142	41.50
Marriage status	Unmarried	83	24.50
	Married	260	75.50

The analysis conducted for gender, age, and education level showed the presence of significant mean differences among these categories (Table 2). Meanwhile, marital status did not significantly correlate with the difference in mean GPH and GMH between married and unmarried categories evaluated [17–21].

**Table 2:** GPH and GMH mean differences among categories

Total score	Category	Number (participants)	Percentage	Mean (SD)	Statistical test	*p*-value
	**Gender**					
Total	Women	226	65.9	12.5 (2.6)	t(340) = -2.5	0.010*
GPH	Men	117	34.1	13.3 (2.8)		
Total	Women	226	65.9	11.9 (2.6)	t(340) = -4.4	0.000*
GMH	Men	117	34.1	13.3 (2.8)		
	**Marital status**					
Total	Married	260	75.5	12.7 (2.6)	t(134.5) = -0.7	0.481
GPH	Unmarried	83	24.5	12.9 (2.7)		
Total	Married	260	75.5	12.4 (2.6)	t(124.9) = 0.0	0.981
GMH	Unmarried	83	24.5	12.4 (3.0)		
	**Education**					
Total	Base	27	7.9	11.5 (2.0)	f(2.340) = 7.9	0.000*
GPH	Intermediate	174	50.6	12.5 (2.4)		
	High	142	41.5	13.4 (2.8)		
Total	Base	27	7.9	11.6 (1.8)	f(2.340) = 12.5	0.000*
GMH	Intermediate	174	50.6	11.8 (2.6)		
	High	142	41.5	13.2 (2.8)		
	**Age group**					
Total	18–24	52	15.2	12.9 (2.3)	f(6.336) = 4.11	0.000*
GPH	25–34	93	27.1	13.4 (2.8)		
	35–44	49	14.3	13.6 (2.8)		
	45–54	74	21.6	12.3 (2.6)		
	55–64	49	14.3	11.8 (2.2)		
	65–74	21	7.9	11.9 (2.4)		
	> = 75	5	1.4	11 (1.4)		
Total	18–24	52	15.2	12.4 (2.9)	f(6.336) = 3.82	0.000*
GMH	25–34	93	27.1	12.5 (2.9)		
	35–44	49	14.3	13.8 (2.6)		
	45–54	74	21.6	11.6 (2.5)		
	55–64	49	14.3	12.2 (2.4)		
	65–74	21	7.9	11.4 (2.1)		
	> = 75	5	1.4	12.6 (2.1)		

*GPH* Global Physical Health, *GMH* Global Mental Health

^*^ = significant (*p* < 0.05);

The content validity in the form of S-CVI from PROMIS Global Health Scale v1.2 was assessed using two different methods. The first was conducted by dividing the number of items with a relevance score of 3–4 from experts by the total items (S-CVI/UA = 0.90) (Table 3). The second was performed through the division of the total I-CVI by the total number of items (S-CVI/AVE = 0.98), with Table 3 presenting I-CVI calculation. The analysis carried out showed that the content validity of PROMIS-GH scale was good due to S-CVI value being > 0.8.

**Table 3:** The analysis of validity evidence based on text content

*Items*	Expert ratings					*Number in Agreement*	I-CVI
	*Expert 1*	*Expert 2*	*Expert 3*	*Expert 4*	*Expert 5*		
Global03	4	4	3	4	4	5	1
Global06	4	4	4	4	4	5	1
Global07r	4	4	3	**2**	3	4	0.8
Global08r	4	4	3	4	3	5	1
Global02	4	4	3	4	4	5	1
Global04	4	4	4	4	4	5	1
Global05	4	4	4	4	4	5	1
Global10r	4	4	4	4	4	5	1
Global01	4	4	3	4	4	5	1
Global09r	4	4	4	3	4	5	1
**Mean I-CVI**							0.98
**S-CVI/UA**							0.90
*Expert Proportion*	1.00	1.00	1.00	0.90	1.00	**Mean Expert Proportion**	0.98

*I-CVI* Item-Content Validity Index, *S-CVI* Scale-Content Validity Index, *S-CVI/UA* Scale-Content Validity Index/Universal Agreement

**Fig. 2:**
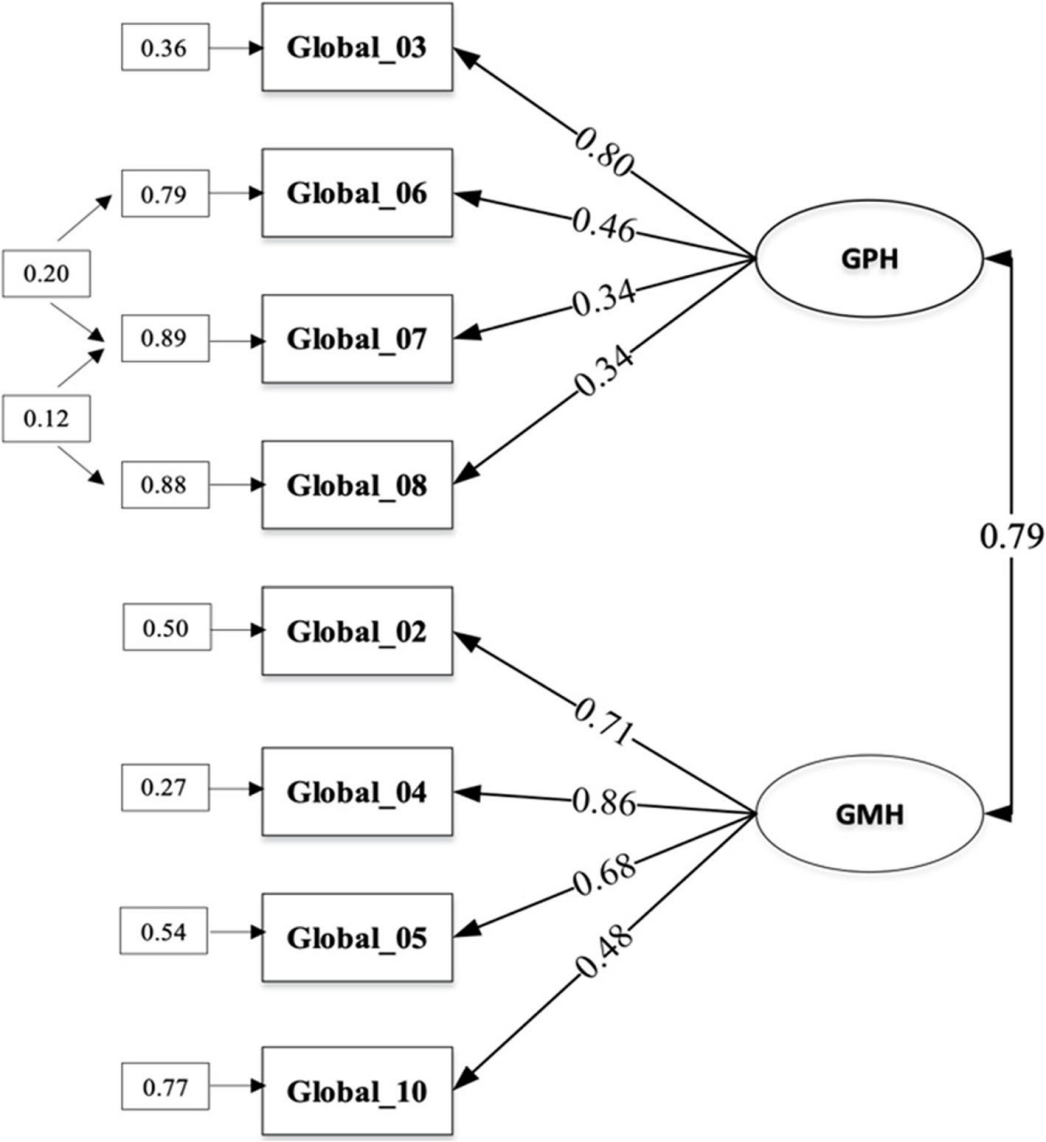
The factor loadings of GPH and GMH

Structural validity was assessed using CFA method to determine the relationship between question items as well as GPH and GMH constructs (Table 4). The hypothesized structural model consisted of a first-order factor with two correlated dimensions (Fig. 2). The result of this model showed a good fit with acceptable fit indices, including RMSEA ≤ 0.08 and CFI > 0.95. Subsequently, test–retest reliability was determined from the intraclass correlation results of pre-test and post-test total scores with values of 0.72 for GPH (95% CI, [0.65,0.78]) and 0.70 for GMH (95% CI [0.63,0.76]).

**Table 4:** The structural validity based on CFA

Measurement	*Two factors score*	Interpretation
${\chi }^{2}/$ *df*	19.90 (13)	Good fit
*p-value*	0.10	
RMSEA	0.04	Good fit
RMR	0.03	Good fit
NFI	0.98	Good fit
CFI	0.99	Good fit

*RMSEA* Root Mean Square Error of Approximation, *RMR* Root Mean Square Residual, *NFI* Normed Fit Index, *CFI* Comparative Fit Index

According to Table 5, the conducted item analysis showed that all items in the measurement instrument had a satisfactory correlation value ≥ 0.30, signifying adequate internal consistency. Cronbach's Alpha of GPH is 0.61 with SD 2.64 and SEM calculated 1.63, while GMH is 0.77 with SD 2.73 and SEM calculated 1.31.

**Table 5:** The results of Cronbach's Alpha coefficient and item analysis of GPH and GMH

	*Reliability* *(Cronbach's Alpha)*	*Corrected Item-Total Correlation*	*Cronbach's Alpha when an item is deleted*
**GPH**	0.61		
Global03		0.46	0.49
Global06		0.43	0.51
Global07 recorded		0.42	0.51
Global08		0.30	0.61
**GMH**	0.77		
Global02		0.58	0.71
Global04		0.71	0.63
Global05		0.58	0.72
Global10		0.44	0.79

*GPH* Global Physical Health, *GMH* Global Mental Health

Evidence of the validity of the internal structure was obtained with CFA using the maximum likelihood estimation method. According to Fig. 2, correlation tests were conducted to determine the relationship between sub-dimensions (latent variables). The results showed that GPH sub-dimension had a good correlation with GMH (r = 0.79). Factor loadings which could be observed from the number in the middle of the arrow showed the correlation between items and sub-dimensions. A value exceeding 0.30 represented a moderate correlation [22], where all items had scores that significantly correlated with GPH or GMH sub-dimensions.

## Discussion

The results in the form of S-CVI showed that the content validity of PROMIS Global Health Scale v1.2 was good (S-CVI > 0.8). In addition, S-CVI calculation signified the ability of the items from this questionnaire to measure the validity construct. Some input from experts included changes to sentence structure, where Global03 was transformed into “How would you rate your general physical health?”. Items Global07r and Global08r were suggested to be changed to “How would you rate your pain on average?” and “How to assess your average fatigue?”. There was an additional input suggested for item Global07r, which was the alteration of the word "nyeri" to "sakit". Despite the input provided by experts, the results of the validity evidence based on the internal structure of PROMIS Global Health Scale were classified as good, leading to the items remaining unchanged.

CFA model showed that GPH and GMH constructs could be measured optimally (valid) by items of PROMIS Global Health Scale v1.2. Fit calculations were performed to determine the model goodness of fit using the chi-square ratio with degrees of freedom (χ2/*df*, CFI, root mean square residual (RMR), and RMSEA. A good model fit was signified by χ2/*df* ≤ 3, CFI close to 1, RMR < 0.08, and RMSEA < 0.05. The results showed an excellent fit with χ2/*df*of 1.53, RMSEA of 0.04, RMR of 0.03, and CFI of 0.99. These were consistent with similar studies using the Hungarian population (GPH: RMSEA = 0.008, SRMR = 0.045, CFI = 0.968, GMH: RMSEA = 0.012, SRMR = 0.031, CFI = 0.990) and the Dutch population (GPH: SRMR = 0.04, GMH: SRMR = 0.03) [23, 24]. A strong correlation between the sub-dimensions of GPH and GMH (*r* = 0.79) was also found, which was consistent with the initial PROMIS study conducted in the United States population (*r* = 0.63) [5].

Reliability testing was conducted with two methods, and the first included assessing internal consistency using Cronbach's Alpha which had satisfactory values of 0.61 for GPH and 0.77 for GMH, respectively. Test–retest reliability was obtained from the intraclass correlation results of pre-test and post-test total scores with moderate to good reliability for both GPH and GMH, thereby signifying the consistency of the instrument assessment outcomes across repeated measurements [22, 25]. A measurement instrument was considered to have acceptable reliability when Cronbach's Alpha value was > 0.7 and the intraclass correlation was > 0.70. The coefficient of correlation between GPH and GMH was 0.79, which was close to the reliability results of 0.81 for GPH and 0.86 for GMH previously obtained from PROMIS Global Health Scale v1.2 development studies. These were consistent with the psychometric assessment study of the Dutch-Flemish PROMIS Global Health Scale v1.2 translation which showed good reliability (GPH 0.78, GMH 0.83) [5].

Table 5 showed that the results of the item analysis on Global08r item were the smallest with a value of 0.3, and this loading factor is smaller than Global03 and Global06. Global08r item assessed the average feeling of fatigue, where physical fatigue could be interpreted as the participants feeling mentally tired. Study by Hays et al. also found that Global08 along with Global07 and Global 10 had the lowest item information [5].

Cronbach's Alpha assessment conducted during the removal of items from GPH showed a decrease in correlation coefficients across all items, while the deletion of Global10 from GMH caused a slight increase in correlation coefficients (0.77 to 0.79), as similarly observed in a previous study. Global10 item assessed the presence of emotional problems and the extent of distress initiated, while the weakness of items could be caused by the emergence of two meanings of the score. A low score could denote that the participants lack emotional problems, or had emotional problems without feeling disturbed.

The results of this study have practical relevance in several ways, the excellent model fit shows that the Indonesian version of PROMIS Global Health Scale v1.2 is valid for measuring GPH and GMH constructs. This may provide healthcare professionals and researchers in Indonesia with an alternative tool for assessing these dimensions of HRQoL. The consistency with results from Hungarian and Dutch population highlights the cross-cultural adaptability of PROMIS Global Health Scale. This ensure the tool’s realibiliy across different cultural context, making it suitable for global studies involving diverse populations. The strong correlation between GPH and GMH sub-dimension, comaparable to finding in the U.S population, suggests that the instrument effectively captures the interrelation between physical and mental health. This support its use in monitoring patient outcomes, evaluating interventions, and conduction population health studies. Given its demonstrated validity and reliability, the tool can be used in HRQoL assessments, addressing the need for less time consuming instruments in public health and clinical practice.

Strengths of the study included a comprehensive psychometric evaluation covering multiple aspects of validity and reliability. The study has several limitations. Despite translation and cultural adaptation, subtle cultural differences in health perceptions might limit applicability to other cultural groups. The study focused on individuals involved in physical medicine and rehabilitation, which may not reflect the broader Indonesian population or other countries. Additionally, validity assessments with other HRQoL instruments, such as SF-36 and SF-12, were not conducted. Patients were not categorized by diagnosis, which may lead to differing health perceptions. The cross-sectional design limits the evaluation of changes over time. Further studies involving a more representative sample of the general population are necessary. Additional cultural and linguistic testing in other countries can confirm the scale’s universal applicability. Conducting studies to evaluate sensitivity to changes over time will enhance understanding of its generalizability in different context.

## Conclusion

In conclusion, this study confirms that the Indonesian version of PROMIS Global Health Scale v1.2 has strong content validity, structural validity, internal consistency, and reliability. It is ready for use in HRQoL assessments in Indonesia, demonstrating effectiveness in both validity and reliability. Consistent results across different populations emphasize its potential to standardize HRQoL measurement globally while meeting local cultural needs, making it an essential tool for advancing health assessments in Indonesia and beyond.

## Data Availability

https://docs.google.com/document/d/1YKyiqcByhVrT--oDHc3voyemcmwb3Myk/edit?usp=drive_link&ouid=118351738973899332756&rtpof=true&sd=true.
